# Lutein and Zeaxanthin Isomers Protect against Light-Induced Retinopathy via Decreasing Oxidative and Endoplasmic Reticulum Stress in BALB/cJ Mice

**DOI:** 10.3390/nu10070842

**Published:** 2018-06-28

**Authors:** Minzhong Yu, Weiming Yan, Craig Beight

**Affiliations:** 1 Department of Ophthalmic Research, Cole Eye Institute, Cleveland Clinic Foundation, Cleveland, OH 44195, USA; ywming@fmmu.edu.cn (W.Y.); Beightc2@ccf.org (C.B.); 2 Department of Ophthalmology, Cleveland Clinic Lerner College of Medicine of Case Western Reserve University, Cleveland, OH 44195, USA; 3 Department of Clinical Medicine, Faculty of Aerospace Medicine, Key Laboratory of Aerospace Medicine of the National Education Ministry, Fourth Military University, Xi’an 710032, China; 4 Louis Stokes Cleveland Veterans Affairs Medical Center, Cleveland, OH 44195, USA

**Keywords:** lutein, RR-zeaxanthin, mesozeaxanthin (RS zeaxanthin), light damage, photoreceptor degeneration, oxidative stress, endoplasmic reticulum stress, electroretinography

## Abstract

Oxidative stress (OS) and endoplasmic reticulum stress (ERS) are the major factors underlying photoreceptor degeneration. Lutein, RR-zeaxanthin (3R,3’R-zeaxanthin) and RS (meso)-zeaxanthin (3R,3’S-RS- zeaxanthin) (L/Zi) could protect against cell damage by ameliorating OS in retina. In this study, we examined the effect of L/Zi supplementation in a mouse model of photoreceptor degeneration and investigated whether the treatment of L/Zi ameliorated OS and ERS. BALB/cJ mice after light exposure were used as the animal model. The protective effects of L/Zi were observed by electroretinography (ERG) and terminal deoxyuridine triphosphate nick-end labeling (TUNEL) analysis. The underlying mechanisms related to OS and ERS were explored by Western blotting. After L/Zi treatment, the ERG amplitudes were significantly higher, and the number of TUNEL-positive cells was significantly reduced compared to that of the vehicle group. Western blotting results revealed that OS was ameliorated according to the significant downregulation of phosphorylated c-Jun N-terminal kinase (p-JNK), and significant upregulation of nuclear factor erythroid 2-related factor 2 (Nrf2). In addition, ERS was reduced according to the significant downregulation of 78 kDa glucose-regulated protein (GRP78), phosphorylated protein kinase RNA-like endoplasmic reticulum kinase (p-PERK), activating transcription factor 4 (ATF4) and activating transcription factor (ATF6). Our data shows that L/Zi provided functional and morphological preservation of photoreceptors against light damage, which is probably related to its mitigation of oxidative and endoplasmic reticulum stress.

## 1. Introduction

Excessive light exposure could often result in photoreceptor degeneration [1]. Accumulated light-induced damage is related to the development of age-related macular degeneration (AMD) [2], a globally prevalent retinal disease that causes blindness [3]. Although the details of the pathogenesis of light-induced retinal damage remain unclear, light-induced oxidative stress is known to cause photoreceptor loss [4,5]. During light exposure, the production of reactive oxygen species (ROS) is increased which causes retinal degeneration [6]. Quite a few antioxidants have been confirmed to be effective in reducing photoreceptor degeneration in animal models of light-induced damage [7,8].

In addition to the role of oxidative stress (OS) involved in the pathogenesis of photoreceptor degeneration, endoplasmic reticulum stress (ERS) also contributed to retinal degeneration in a variety of conditions such as age-related macular degeneration [9,10], retinitis pigmentosa (RP), diabetic retinopathy [11,12], and glaucoma [13,14]. Light exposure was also reported to induce ERS as well as abnormal ER membranes and endomembranes in 661W cells [15]. In addition, the levels of ERS protein markers are up-regulated in mouse retinas following exposure to light [16]. Consistent with this hypothesis, a number of studies have demonstrated that early administration of the agents that inhibit ERS could significantly decrease the rate of photoreceptor cell death in animal models of light-induced retinopathy [15,17].

Some carotenoids are highly concentrated in the light-exposed structures in plants and in the human retina [18]. Lutein, zeaxanthin and mesozeaxanthin (L/Zi) belong to the class of xanthophyll carotenoids, which are found in a number of fruits and vegetables [19] and are the major carotenoids in the human retina [20,21]. Higher dietary lutein and zeaxanthin intake can reduce the incidence of AMD, while low level of L/Zi has been associated with AMD [22]. Furthermore, administration of L/Zi has been showed to protect against retinal cell damage in diabetic retinopathy [23]. Barker et al. [24] reported that lutein or zeaxanthin supplementation protected the fovea against acute blue light-induced retinal damage in rhesus monkeys, which is mainly attributed to the anti-oxidative properties of L/Zi [18,25]. However, the mechanisms of ERS underlying the effect of L/Zi in the amelioration of light-induced retinopathy have not been fully elucidated [21]. The BALB/cJ mouse exposed to light is a commonly used model characterized by photoreceptor degeneration [26]. The purpose of this study is to investigate the protective effects of L/Zi supplementation on this mouse model of early retinal cell degeneration and the underlying mechanisms of OS and ERS.

## 2. Materials and Methods

### 2.1. Animals and Experiment Design

Male BALB/cJ mice (9–13 weeks old) (Stock Number: 000651) were purchased from the Jackson Laboratory (Bar Harbor, ME, USA) and were housed under the same conditions in a low-illuminance (extracage/intracage: 13 lx/1 lx) vivarium under cyclic light (14 h light and 10 h dark) [27]. All animal experiments were performed in accordance with the Association for Research in Vision and Ophthalmology (ARVO) Statement for the Use of Animals in Ophthalmic and Vision Research, and were approved by the Institutional Animal Care and Use Committee of the Cleveland Clinic Foundation (IACUC protocol #2013-0933).

L/Zi, (10 mg/kg of body weight, OmniActive Health Technologies Ltd., Maharashtra, India) dissolved in sunflower oil (1 mg/mL, SFO, Sigma, St. Louis, MO, USA) or equal volume of SFO as vehicle was administered by daily oral gavage to BALB/cJ mice in treatment group (*n* = 7) and vehicle group (*n* = 7), respectively for a 5-day period from Day 1 to Day 5. The dose of L/Zi was chosen after a preliminary experiment (Data not shown), which is the maximum dose without the adverse effect on body weight. The number of the animals in our study was conservative enough to achieve the statistical power of at least 0.8.

### 2.2. Light Exposure

Mice were dark-adapted for 12 h, and then the pupils were dilated with 1% Tropicamide Ophthalmic Solution (Bausch & Lomb, Rochester, NY, USA). Mice were exposed to blue light (5000 lx) for 1 h. The blue light was obtained by filtering white fluorescent light by a filter which transmits light between 380 and 570 nm (Midnight Blue 5940, Solar Graphics, Clearwater, FL, USA). The mice were free to move in the light chamber during the light exposure. After the light exposure, the mice were returned to the low-illuminance animal room where they were housed.

### 2.3. Electroretinography (ERG)

After overnight dark adaptation, mice were anesthetized with a mixture of ketamine (80 mg/kg) and xylazine (16 mg/kg) diluted in saline. The pupils were dilated with 1% mydriacyl (tropicamide), 1% cyclopentolate HCl, 2.5% phenylephrine HCl) and the corneal surface was anesthetized with 0.5% proparacaine HCl eye drops. Stimulus response functions were obtained under dark- and then light-adapted conditions, and ERG a-wave and b-wave amplitudes in multiple flash luminances were measured and analyzed using published procedures [28].

### 2.4. TUNEL Analysis and Measurement of the Thickness of Outer Nuclear Layer (ONL)

Apoptosis was detected using the In Situ Cell Death Detection Kit (Roche Applied Science, Indianapolis, IN, USA). After the ERG recording, the eyes of the mice were immediately enucleated under euthanized, and then fixed in 4% paraformaldehyde for 2 h. The eyes were dehydrated in graded sucrose solutions (10–30%) and embedded in Optimal Cutting Temperature (OCT) compound. Retinal sections of 10 µm in thickness were cut near the optic nerve and incubated with freshly prepared 0.1% Triton X-100/0.1% sodium citrate permeabilization solution for 2 min on ice. After rinsing with phosphate buffered saline (PBS) for 3 times, sections were incubated with the TUNEL reaction mixture for 60 min at 37 °C in the dark and then rinsed with PBS 3 times. Sections were mounted with VECTASHIELD mounting medium with 4′,6-diamidino-2-phenylindole (DAPI) (Burlingame, CA, USA), and visualized at 400× magnification with the fluorescence microscope (BX60, Olympus, Tokyo, Japan). The pictures were taken in the area next to the optic nerve head. The number of apoptotic cells was counted in three sections of each eye and averaged.

For the measurement of the thickness of ONL, the DAPI-stained pictures taken in the TUNEL procedure were used. The thickness of the ONL was measured at 200 μm from the edge of the optic disc on either side of the optic nerve head using ImageJ 1.48v software (National Institutes of Health, Bethesda, MD, USA). Four sections from each retina were measured to calculate the mean of ONL thickness of that retina.

### 2.5. Western Blotting

The retinas were collected and homogenized on ice with the protein extraction reagent. Lysates were then centrifuged at 16,000 rpm at 4 °C for 25 min to obtain the supernatant. The protein content of the retinal extracts was measured by Pierce 660 nm Protein Assay Reagent (Thermo Scientific, Rockford, IL, USA). Equal amounts of protein (15 µg) of each extract in Laemmli Sample buffer was heated on a boiling water bath for 7 min and thereafter electrophoresed on 8–16% gradient SDS-polyacrylamide gel. After electrophoresis, proteins were transferred to a polyvinylidenedifluoride (PVDF) membrane. The membrane was blocked with Tris-buffered saline containing 0.1% Tween-20 (TBST) and 5% dried non-fat milk at room temperature for 2 h. Blocked membrane was incubated with indicated primary antibodies (anti-p-JNK1+p-JNK2+p-JNK3, 1:2000, #ab124956; anti-GRP78/BiP, 1:2000, #ab21685; anti-ATF6, 1:1500, #ab37149; Abcam, Cambridge, MA, USA. anti-Nrf2, 1:1000, #sc-722; anti-p-PERK, 1:500, #sc-32577; anti-ATF4, 1:1000, #sc-200; Santa Cruz Biotechnology, Dallas, TX, USA) at 4 °C overnight, followed by incubating with the goat-anti-rabbit horseradish peroxidase-conjugated secondary antibody for 16 h at 4 °C. Membrane was washed 3 times with TBST and then incubated in TBST containing 1:7000 diluted Goat-anti-rabbit IgG - Horseradish Peroxidase (HRP) (sc-2004, Santa Cruz Biotechnology, Dallas, TX, USA) for 1 h at 25 °C. Membrane was washed again with TBS-TW20 (3 times) and antigen-antibody complexes were visualized by the enhanced chemiluminescence-2 (ECL-2, Thermo Scientific, Rockford, CA, USA). Beta-actin (#4970, Cell Signaling Technology, Danvers, MA, USA) was used as internal control.

### 2.6. Statistical Analysis

For the analysis of ERG data, two-way repeated measures Analysis of Variance (ANOVA) was used. The power analysis was conducted by the F-test of one-way ANOVA, where we considered numbers as outcome and groups as the factor. All other comparisons were made by one-way ANOVA. A *p* value of less than 0.05 was considered statistically significant.

## 3. Results

### 3.1. Effect of L/Zi on Retinal Function

**Figure 1:**
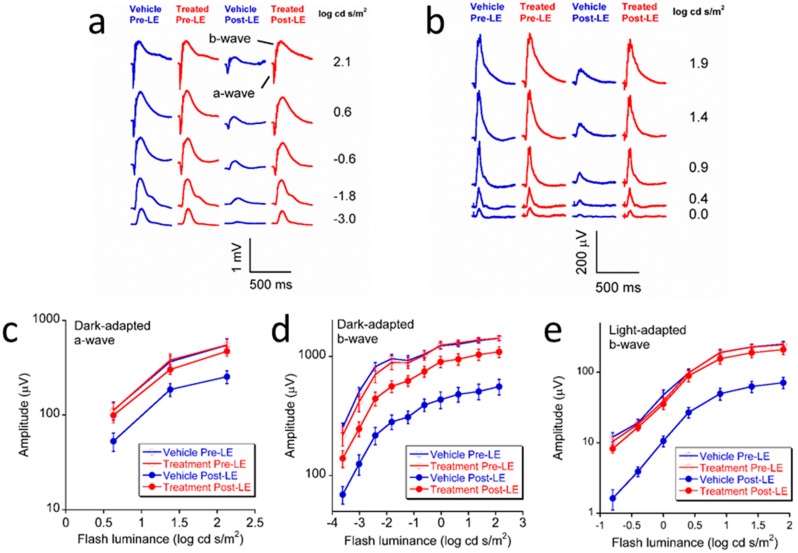
Lutein, RR-zeaxanthin (3R,3’R-zeaxanthin) and RS (meso)-zeaxanthin (3R,3’S-RS- zeaxanthin) (L/Zi) treatment rescued retinal function in light-damaged retinas. Typical waveforms of electroretinogram (ERG) and luminance-response curves of BALB/cJ mice before (9–11 weeks old) and after (11–13 weeks old) blue light exposure in vehicle (sunflower oil) and L/Zi treatment groups (*n* = 7 in each group). (**a**) Typical dark-adapted ERGs waveforms; (**b**) Typical light-adapted ERGs waveforms; (**c**) Luminance-response curves of a-wave amplitudes in dark-adapted conditions which are associated with the responses from rod and cone photoreceptors; (**d**) Luminance-response curves of b-wave amplitudes in dark-adapted condition which are associated with the responses from bipolar cells in both rod and cone pathways; (**e**) Luminance-response curves of b-wave amplitudes in light-adapted condition which are associated with the responses from bipolar cells in the cone pathway. Error bars indicate standard errors. Pre-LE: Before light exposure. Post-LE: After light exposure.

ERG was used to compare outer retinal function of mice. Before the light exposure, the a-wave and b-wave amplitudes of both dark-adapted and light-adapted ERG of the vehicle group and the treatment group were almost the same, with no statistical difference (*p* > 0.05). Intense light exposure led to significant reductions in a- and b-wave amplitudes in vehicle group under both dark-adapted and light-adapted conditions (*p* < 0.05), while the amplitudes were significantly higher in L/Zi treated group under all luminances (all *p* < 0.01). In addition, our data shows that L/Zi treatment ameliorated the decrease of light-adapted ERG b-wave amplitude more markedly than that of the dark-adapted ERG b-wave amplitude, indicating that the protective effect of L/Zi to the cone system was better than to the rod system (Figure 1).

### 3.2. Effect of L/Zi on Cellular Apoptosis

**Figure 2:**
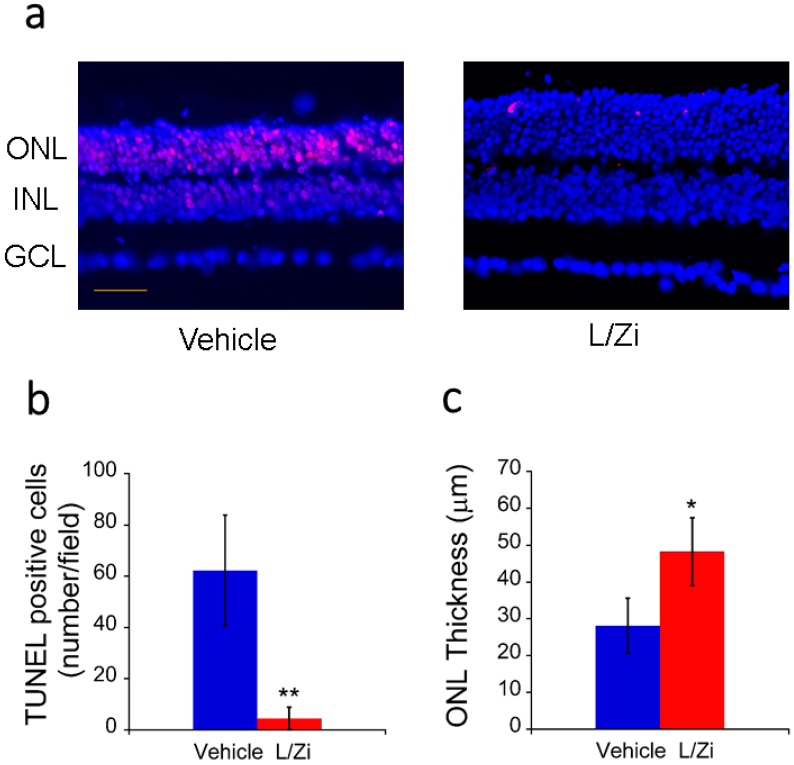
L/Zi treatment reduced cellular death in light-damaged retinas. (**a**) Representative images (20×) of the staining with TUNEL assay. The dead cells are shown as red spots. Scale bar: 30 µm; (**b**) Means of the number of TUNEL-positive cells in retinal sections of BALB/cJ mice treated with vehicle or L/Zi (*n* = 3 in each group); (**c**) Means of the ONL thickness in retinal sections of BALB/cJ mice treated with vehicle or L/Zi (*n* = 3 in each group). ONL: outer nuclear layer; INL: inner nuclear layer; GCL: ganglion cell layer. Error bars: Standard deviations. * *p* < 0.05 vs. vehicle group. ** *p* < 0.01 vs. vehicle group.

TUNEL assay was performed on retinal sections to examine the TUNEL-positive cells, which include apoptotic cells [29] and different types of dying cells [30]. In the light-damaged retinas, significantly more TUNEL-positive cells were found in the retinal sections of vehicle group, predominantly in the outer nuclear layers (ONL). In comparison, TUNEL-positive cells were scarce in the retina of mice in L/Zi treatment group. Quantitative analysis revealed a significant difference of TUNEL positive cells in retinal section between vehicle and treatment group after light damage (vehicle group versus treatment group, *p* < 0.01) (Figure 2a,b).

### 3.3. Effect of L/Zi on Outer Nuclear Layer Thickness

The ONL thicknesses were measured in the DAPI-stained images. In the light-damaged retinas, the ONL thickness was less in the vehicle group than that in L/Zi treatment group. Quantitative analysis revealed a significant difference of ONL thickness in retinal section between vehicle and treatment group after light damage (vehicle group versus treatment group, *p* < 0.05) (Figure 2c).

### 3.4. Effect of L/Zi on OS in Light-Damaged Retinas

**Figure 3:**
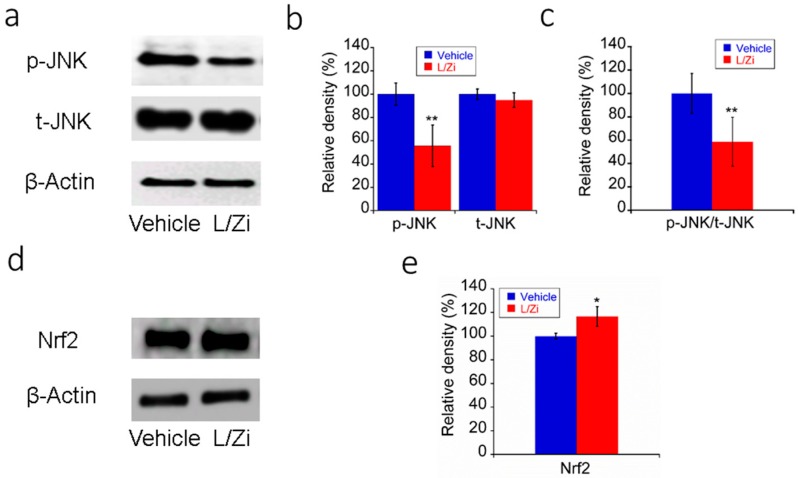
L/Zi treatment down-regulated the markers of oxidative stress in light-damaged retinas. (**a**) Representative images of Western blotting of phosphorylated c-Jun N-terminal kinase (p-JNK); (**b**) Relative protein expression of p-JNK; (**c**) Ratio of protein densities of p-JNK to total (t)-JNK in light-damaged mice (*n* = 3 in each group); (**d**) Representative images of Western blotting of Nuclear factor (erythroid-derived 2)-like 2 (Nrf2); (**e**) Relative protein density of Nrf2 in light-damaged mice (*n* = 3 in each group). * *p* < 0.05 vs. vehicle group; ** *p* < 0.01 vs. vehicle group.

C-Jun N-terminal kinase (JNK) and Nuclear factor (erythroid-derived 2)-like 2 (Nrf2) were chosen as protein markers of OS in our study. Western blotting results showed that L/Zi treatment significantly downregulated p-JNK and reduced the ratio of the protein densities of p-JNK to t-JNK (vehicle group versus treatment group, all *p* < 0.01), while the t-JNK were not changed significantly between the two groups (*p* > 0.05). In addition, L/Zi significantly upregulated Nrf2 in light-damaged retinas (vehicle group versus treatment group, *p* < 0.05) (Figure 3).

### 3.5. The Effect of L/Zi on Light-Induced ERS

**Figure 4:**
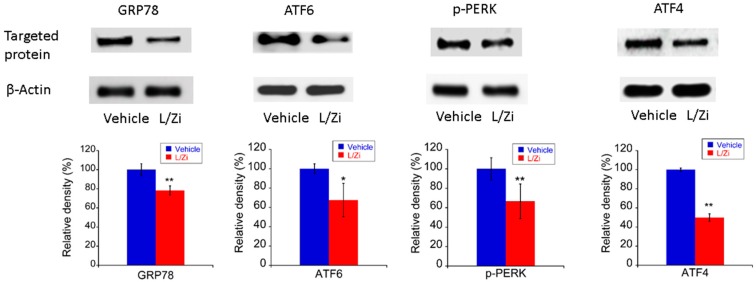
L/Zi treatment down-regulated the markers of ERS in light-damaged retinas. Representative images of Western blotting and relative protein expression of 78 kDa glucose-regulated protein (GRP78), activating transcription factor 6 (ATF6), phosphorylated protein kinase RNA-like endoplasmic reticulum kinase (p-PERK) and activating transcription factor 4 (ATF4) in light-damaged retinas, respectively (*n* = 3 in each group). Error bars indicate standard deviations. * *p* < 0.05 vs. vehicle group; ** *p* < 0.01 vs. vehicle group.

In our study, the expression levels of four ERS protein markers, including 78 kDa glucose-regulated protein (GRP78), activating transcription factor 6 (ATF6), phosphorylated protein kinase RNA-like endoplasmic reticulum kinase (p-PERK) and activating transcription factor 4 (ATF4), were determined by Western blotting to investigate whether L/Zi treatment reduced ERS in light-damaged retinas. Western blotting results revealed the expression of these ERS markers were significantly reduced by L/Zi treatment (vehicle group versus treatment group: GPR78, *p* < 0.01; ATF6, *p* < 0.05; p-PERK, *p* < 0.01; ATF4, *p* < 0.01) (Figure 4).

## 4. Discussion

Animal models of light-induced retinopathy have been used to study phototoxic retinal damage and associated mechanisms, including oxidative stress responses and metabolic abnormalities [31,32,33,34]. It was observed that many genes were upregulated in light-damaged retinas of Balb/c mice.

The upregulated genes are in the categories of anti-oxidants, anti-apoptosis, chloride channels, transcription factors, secreted signaling molecules, and inflammation. The upregulated genes may affect the fate of photoreceptors by photo-oxidative stress [35]. A number of studies have shown that OS is one of the main factors which causes light-induced disruption of photoreceptor outer segment and retinal degeneration [36,37,38,39]. In addition, the aggregation of S-opsin and ERS was observed in light-damaged models in vitro and in vivo [40,41,42]. More and more evidence shows that the activation of unfolded protein response on exposure to oxidative stress is an adaptive mechanism to preserve cell function and survival [43]. Reactive oxygen species (ROS), produced in the ER or other organelles, can target the ER calcium channels which causes the release of calcium from ER. The calcium is then obtained by mitochondria, which increases the mitochondrial metabolism and production of ROS. ROS can trigger ER stress [44]. Both of OS and ERS could finally induce cellular apoptosis. Antioxidants with the abilities of ameliorating the OS or ERS have been reported to reduce the light-induced damage on retinas [7,15]. The xanthophyll carotenoids—lutein, zeaxanthin and mesozeaxanthin (L/Zi) have higher concentration in visual system (eye and brain) compared to other carotenoids in the blood [21]. Specifically, L/Zi comprise 80% to 90% of xanthophyll carotenoids in human eyes [20], and decreased level of these antioxidants has been associated with AMD [25]. In addition, administration of L/Zi has been showed to protect against retinal cell damage in many eye pathologies, such as diabetic retinopathy [23].

In our study, the protective effect of L/Zi on retina was observed in an animal model of retinal degeneration induced by extensive blue light exposure, which was confirmed by the significant improvement of dark-adapted and light-adapted ERG a-wave and b-wave amplitudes, and reduction of the number of TUNEL-positive cells in the retinas. We further explored the effect of L/Zi on protein markers of OS to understand the exact underlying mechanism of cellular protection provided by L/Zi. c-Jun N-terminal kinase (JNK) is one the three well-defined subgroups of mitogen-activated protein kinase (MAPK) [45]. The activation of JNK induces a cascade of phosphorylation events, leading to the formation of phosphorylated (p)-JNK, which has been associated to the OS [46]. Nuclear factor (erythroid-derived 2)-like 2 (Nrf2) is a transcription factor recognized as a pivotal element of cellular defense responses to an increased level of ROS [47]. In an inactive status, Nrf2 protein binds to Keap1 [48]. When Nrf2 is activated, Nrf2 separates from Keap1 and moves into the cell nucleus by phosphorylation of Nrf2. The released Nrf2 then binds with antioxidant response element to promote the expression of phase II enzymes and endogenous antioxidants to restore the homeostasis of reactive oxygen species [49,50]. Our Western blotting data of the light-damaged retinas treated with L/Zi showed that p-JNK expression was significantly downregulated. This is consistent with the finding that lutein and zeaxanthin downregulated the lipopolysaccharide-induced increase of p-JNK levels in cultured human uveal melanocytes [51]. Furthermore, our Western blotting data also revealed that the Nrf2 expression was significantly upregulated in mice with L/Zi treatment, which is consistent with the study in mouse microglial (BV-2) cells [52]. These data implied that the MAPK and Nrf2 pathways, which are involved in OS response, may be positively and negatively related to the photoreceptor degeneration in light-damaged retina, and the treatment effect of L/Zi may be related to the regulation of these cellular pathways.

ERS results from protein misfolding in the ER, which has interaction with OS [44,53] and causes cell death [54]. Its role in the light-induced retinal damage has also been illustrated [40]. GRP78, p-PERK and ATF6 are part of protein markers of ER stress. Specifically, GRP78 is a master regulator of the unfolded protein response in ER [55]. PKR-like ER kinase (PERK) is one of the major signaling pathways of ERS, which regulates cellular protein synthesis related to the influx of proteins into the lumen of the stressed ER [56,57]. ATF4 is a downstream transcription factor in PERK signaling pathway. Activated ATF4 increases CCAAT-enhancer-binding protein homologous protein (CHOP) expression, which in turn regulates the expression of a number of stress-induced target genes and amplify the signal initiated by the original stress [58]. ERS can also activate the Activating Transcription Factor 6 (ATF6) which optimizes protein folding and degradation [59,60,61]. The Western blotting results in our study demonstrated that L/Zi reduce ERS in light-damaged retinas by downregulating key protein markers of ERS, including GRP78, p-PERK and ATF6 pathway. A similar reduction of these ERS biomarkers was observed in diabetic mice treated by wolfberry with high content of lutein and zeaxanthin [62]. Our data indicated that L/Zi might exert its protective effects at least partially through reducing ERS.

## 5. Conclusions

Treatment with L/Zi could protect photoreceptors against degeneration induced by high intensity of blue light. The treatment effect of this xanthophyll carotenoid is probably related to the decrease of OS and ERS pathways. Future studies are needed to explore this study of L/Zi in other animal models of inherited retinal degeneration, which could form the basis of clinical trials of different types of retinal degenerations that affect humans.
